# Different therapeutic approaches on quality of life in patients with inflammatory bowel disease

**DOI:** 10.1186/s12876-014-0199-5

**Published:** 2014-11-25

**Authors:** Junjie Xu, Hui Lin, Xu Feng, Minyue Tang, Jun Shen, Zhihua Ran

**Affiliations:** Division of Gastroenterology and Hepatology, State Key Laboratory for Oncogenes and Related Genes, Key Laboratory of Gastroenterology &Hepatology, Ministry of Health, Ren Ji Hospital, School of Medicine, Shanghai Jiao Tong University, Shanghai Cancer Institute,Shanghai Institute of Digestive Disease, 145 Middle Shandong Road, Shanghai, 200001 China; Department of General Surgery, Sir Run-Run Shaw Hospital, Zhejiang University, Hangzhou, China; Department of Reproduction, Women’s Hospital, Zhejiang University School of Medicine, Hangzhou, 310006 China

**Keywords:** Inflammatory bowel disease, Health-related quality of life, Inflammatory bowel disease questionnaire, Infliximab

## Abstract

**Background:**

The chronic nature of inflammatory bowel disease leads to considerable impairment on the health related quality of life (HRQOL). The aims of the present study are to validate the mainland Chinese translation of the Inflammatory Bowel Disease Questionnaire (MCIBDQ), and to evaluate the impact of infliximab treatment on HRQOL in patients with IBD for the first time in China, as compared with other therapies of different levels. Furthermore, the impact of different medical therapies on marriage, employment and economic burden in IBD patients were also evaluated.

**Methods:**

Consecutive patients who met inclusion/exclusion criteria were investigated with MCIBDQ, SF-36, disease activity index (DAI), marriage, employment and economic burden questionnaires before and after treatment.

**Results:**

MCIBDQ showed significant reliability and validity both in CD and UC patients. The scores of total SF-36, total MCIBDQ and all domains were found significantly increased, while both DAI and health transition on general health scores were found significantly decreased after infliximab treatment (all *P* < 0.001). Scores of SF-36 and MCIBDQ increased significantly more in infliximab group than non-infliximab group (all *P* < 0.05). Infliximab treatment was suggested to significantly reduce the negative impact on love (*P* = 0.037), increase work time (*P* = 0.016) and ease economic burden (*P* = 0.048).

**Conclusions:**

MCIBDQ was demonstrated to be a reliable and valid scale applied in Chinese IBD patients. Infliximab treatment was found to significantly improve HRQOL in IBD patients in comparison with conventional treatments. Negative impact on marriage, employment, and economic status was found in patients with IBD.

**Electronic supplementary material:**

The online version of this article (doi:10.1186/s12876-014-0199-5) contains supplementary material, which is available to authorized users.

## Background

Inflammatory bowel disease (IBD) is an immune-mediated, chronic disorder characterized by remittent or progressive inflammation that may affect the entire gastrointestinal tract, with two major clinical forms: Crohn’s disease (CD) and ulcerative colitis (UC) [1]. The natural history of the lesions in the alimentary tract, together with the complications, frequent physician visits, associated hospitalizations, and even the adverse events of medical therapy or surgery, lead to considerable impairment on the health related quality of life (HRQOL) in patients with IBD [2]. Recent studies have indicated that patients with active disease suffered poorer HRQOL than patients with quiescent disease [3,4]. Thus, the effective goals of treatment were not only relieving symptoms, diminishing disease activity, or delaying progression, but also improving the patients’ quality of life [5]. Therefore, improvement of patient’s HRQOL became one of the fundamental goals of IBD treatment [6].

Infliximab (IFX) is a genetically engineered monoclonal tumor necrosis factor-alpha (TNF-α) antibody that represents the first effective biologic therapy, which greatly improves the treatment options in managing IBD [7]. It has already been reported that infliximab had a considerable efficacy on induction and maintenance of remission in refractory patients with CD and UC [8,9]. Recent studies on the impact of infliximab on HRQOL in patients with IBD found that anti-TNF therapy was effective on improving HRQOL [10,11], while traditional medications showed controversial effects on patients’ HRQOL [12]. However, these findings were not widely accepted.

When studies evaluating HRQOL were performed, Inflammatory Bowel Disease Questionnaire (IBDQ) was widely applied for HRQOL assessment in patients with IBD, and was proven to be valid and reliable [13]. IBDQ was translated into different languages and validated for each adaption [14,15]. In eastern countries, the incidence of IBD used to be much lower than that of Western countries, but experienced a significant increase in recent decades, making IBD no longer an uncommon disease [16]. However, studies concerning the effect of infliximab treatment on IBD patients’ HRQOL have not been reported in China to date.

The present study aimed to validate the Chinese version of IBDQ, evaluate and compare the impact of infliximab therapy and other different therapies on HRQOL in patients. Furthermore, the impact of infliximab therapy on marriage, employment, and economic burden in IBD patients were also evaluated.

## Methods

### Patients

A total of 119 consecutive patients with IBD (84 with CD and 35 with UC) hospitalized at Renji hospital from Jan 2011 to June 2012 were enrolled in this study. Each of them fulfilled the inclusion criteria, which included: definite diagnosis of CD or UC according to the “World Gastroenterology Organization Practice Guidelines for the Diagnosis and Management of IBD in 2010” [17]; the ability to comprehend and complete the self-administered questionnaire with or without assistance. The exclusion criteria were the presence of a psychiatric disorder, stoma, malignancy, any condition that apparently affect the quality of life, or refusal to be recruited in this study. This protocol was approved by Research Ethics Committee of Shanghai Jiao-Tong University, School of Medicine, and written informed consent was obtained from all the participants before inclusion.

### Tools of assessment

#### IBDQ, SF-36 and general health status

IBDQ was originally developed by Irvine as a physician-administered disease-specific questionnaire regarding the IBD patient’s status in the previous 2 weeks before administration [13]. Thereafter, the self-administered IBDQ was developed and validated by Irvine and colleagues [18], then Mainland Version of IBDQ (MCIBDQ) was translated and validated by Zhou [15]. In the present study, MCIBDQ was applied. It consisted of 32 questions divided into four domains: bowel symptoms (10 items), systemic symptoms (5 items), emotional function (12 items) and social function (5 items). Every question had graded responses and was scored on a 7-point Likert scale from 1 (worst situation) to 7 (best situation), reflecting the QOL of the subjects in the previous 2 weeks. For validation, HRQOL was also assessed using a widely accepted generic instrument assessing 8 dimensions in life - 36-Item Short Form Health Survey Scale (SF-36) [19], and Chinese version was adapted in this study [20,21]. The details of MCIBDQ and the Chinese version of SF-36 were listed on Additional file 1: Table S1, with higher scores indicating better health status. In SF-36, the single item scale on health transition was scored separately as self-evaluation on the changing of general health status, with lower scores indicating better status.

### Disease activity indices

Avoiding invasive procedures, Harvey–Bradshaw simple index (HBI) [22] was calculated in patients with CD, and Walmsley simple colitis activity index (CAI) [23] was used in patients with UC. These two simplified indices relied entirely on symptoms, and highly correlated with the previous complex indices, making evaluation of disease activity and therapeutic response much easier. Thus, these two indices were widely utilized. In both indices, <4 were indicated as remissions or quiescent and ≧4 were considered as active status.

### Marriage, employment and economic burden

Marriage, employment and economic status are three major impaired aspects after being diagnosed with IBD among patients. Thus, a special scale consisting of six items on general status of marriage, employment and economic burden was developed and used in this study. Scores were calculated by every single item, and each item reflected a certain aspect in life. An additional file showed the content of the scale of marriage, employment and economic burden [see Additional file 2].

### Validation of MCIBDQ

In general, the validation mainly included reliability and validity. The reliability of an instrument can be considered as the degree of consistency of its measurement [24], with two main forms: test-retest reliability and internal consistency. For a medical or health care instrument to be clinically useful, it must possess validity. Validity, the ability of an instrument to assess fully what it was intended to measure, was assessed in 4 ways according to standard definitions including content validity, construct validity, discriminant validity, and criterion validity [25]. Although Leong et al. [26] translated and validated the IBDQ in Hong Kong in 2003 and Zhou YX et al. [15] finished the version in Mainland Chinese in 2007, differences in geographic regions, economy, lifestyle, population source, and sample representation suggested that re-validation of MCIBDQ is needed in the present study. Moreover, with high speed of westernization evolution in Mainland China, lifestyle and health concept saw significant alteration in the patients residing, increasing the necessity for the re-validation of MCIBDQ. Details about the background and process were shown on Additional file 3.

### Study design and statistical analysis

The data collected from the consecutive patients who met all inclusion/exclusion criteria were results of a stratified-controlled and matched prospective cohort study, which was designed to determine the reliability and validity of MCIBDQ, and the impact of infliximab treatment on HRQOL in patients with IBD, as compared with other therapies of different levels. The questionnaires were self-administered in most of the cases. A gastrointestinal physician, who was blinded to the results of MCIBDQ, SF-36 and additional questions (marriage, employment and economic burden), would then interview the subjects upon completion to address any questions or issues completing the questionnaires. This physician then scored the disease activity respectively for CD and UC patients on the basis of HBI and CAI. Only a few individuals were unable to read but could comprehend the questions, and completed the questionnaires with the physician’s help. To measure the daily function and quality of life respectively at the time of diagnosis and 2 weeks after the treatment, participants were asked to complete the same questionnaires twice at an interval of 2 weeks to 2 months. All the scales were scored as recommended by their original inventor.

According to highest therapeutic level the subjects had ever received, the participants who completed the questionnaires twice were stratified into four treatment groups as following: 1)anti-inflammatory drugs (oral or intravenous); 2) glucocorticoids (oral, intravenous or local application); 3) immunosuppressive agents (oral or intravenous); 4) biological agents (intravenous IFX) [27]. According to the times of infliximab infusions (dosage: 5 mg/kg; infusion intervals: administered at weeks 0, 2, and 6, and then every 8 weeks), group 4 could also be stratified into 2 subgroups: *a*) <4 times; *b*) ≥4 times. The effect of different therapies on HRQOL among these four treatment groups was determined and compared on the basis of the “variation of scores” (defined as the latter scores minus the former scores) [28,29].

Furthermore, participants who completed the questionnaires twice were divided into two paired groups (infliximab group and control group) according to IBD subtype, disease activity, disease duration, gender, age, career, literacy and marriage, with every subject matched his/her paired subject. Thus, the impact of different therapies on HRQOL between the paired groups was also assessed. Moreover, general status of marriage, employment and economic burden was described in this study.

Cronbach’s α was determined by all available data excluding missing responses, while criterion validity and discriminant validity were determined by all available data including missing responses, which could be replaced according to the original inventors’ suggestion [30]. Construct validity were determined using the data of fully-completed MCIBDQ results.

## Results

### Demographics and general information

A total of 119 consecutive subjects finished the questionnaires for the first time, and 104 of them completed the questionnaires a second time. The general information of subjects who finished both before and after infliximab infusion was shown on Table 1.

**Table 1:** **General information**

**Items**	**CD**	**UC**	**IBD**
Number	74	30	104
Gender: male/female	54/20	17/13	71/33
Mean age(years ± SD)	30.73 ± 10.38	34.97 ± 11.77	31.95 ± 10.91
Disease duration:≤ 1 year/ ≤5 years /> 5 years	9/44/21	5/17/8	14/61/29
Marital status: married/single/divorced	40/34	20/9/1	60/43/1
Education level: ≤ 12 year/ >12 years	26/48	15/15	41/63
Employment: working/unemployed/student/retired	54/5/14/1	20/4/5/1	74/9/19/2
Highest treatment level:1/2/3/4	6/10/22/36	9/11/5/5	15/21/27/41

### Validation of MCIBDQ

#### Reliability

##### Internal consistency

Internal consistency could be represented by Cronbach’s α (values of >0.7 indicating adequate consistency). Cronbach’s α of the 4 MCIBDQ domains ranged from 0.88– 0.95 in CD and from 0.86–0.94 in UC, and the total Cronbach’s α for MCIBDQ was 0.98 in CD and 0.97 in UC, suggesting that MCIBDQ had a strong internal consistency (Table 2).

**Table 2:** **Details and validation of MCIBDQ**

**Domain**	**Cronbach’s α**				**Discriminant ability**									
	**CD**		**UC**		**CD**					**UC**				
	**N** ^**a**^	**Value**	**N** ^**a**^	**Value**	**Remission**		**Active**		***P*** ^**b**^	**Remission**		**Active**		***P*** ^**b**^
					**N** ^**a**^	**Mean (95% CI)**	**N** ^**a**^	**Mean(95% CI)**		**N** ^**a**^	**Mean (95% CI)**	**N** ^**a**^	**Mean (95% CI)**	
Bowel symptoms	149	0.92	43	0.93	84	58.96 (56.88-61.05)	71	44.52 (41.72-47.33)	<0.001	32	56.11 (51.70-60.52)	33	38.70 (34.74-42.67)	<0.001
Systemic symptoms	155	0.88	65	0.86	85	25.08 (23.82-26.35)	71	17.94 (16.39-19.50)	<0.001	32	23.63 (21.37-25.89)	33	18.00 (15.85-20.15)	<0.001
Emotional function	154	0.95	60	0.94	85	66.63 (63.91-69.35)	71	50.20 (46.67-53.73)	<0.001	32	60.10 (54.34-65.86)	33	45.59 (41.30-49.89)	<0.001
Social function	131	0.88	58	0.89	84	27.46 (25.96-28.97)	70	20.26 (18.44-22.07)	<0.001	32	26.44 (23.64-29.24)	33	17.82 (15.33-20.31)	<0.001
Total	121	0.98	52	0.97	83	177.83 (170.83-184.84)	70	133.52 (124.43-142.41)	<0.001	32	166.28 (152.01-180.55)	33	120.11 (108.30-131.93)	<0.001

^a^Number of valid cases; ^b^ *P*-values of independent samples *t*-test.

#### Validity

##### Content validity

Kaiser-Meyer-Olkin (KMO) Measure of Sampling Adequacy and Bartlett’s Test of Sphericity showed that MCIBDQ possess a significant construct validity in both CD patients (KMO value = 0.938, *P* < 0.001) and UC patients (KMO value = 0.837, *P* < 0.001), suggesting the appropriateness of MCIBDQ for factor analysis.

**Figure 1:**
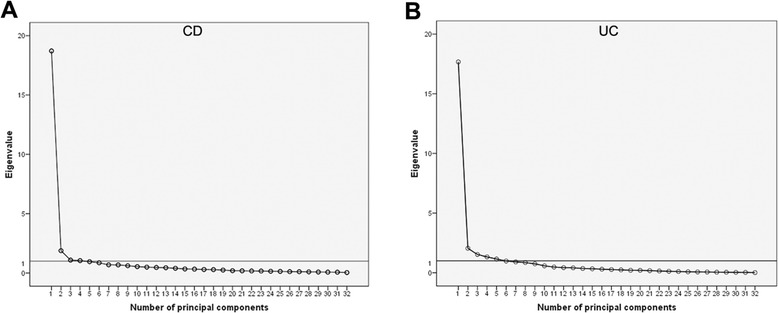
**Scree plots of MCIBDQ from the exploratory factor analysis.** The horizontal line represents the cut-off for selecting the number of factors. **A**, CD patients group; **B**, UC patients group.

In CD patients, explorative factor analysis demonstrated that 4 principal components (each eigenvalue >1) provided 71.04% cumulative contribution of variance, indicating that 4 aggregating dimensions might conveniently summarize the questionnaire, as shown by the scree plot in Figure 1A. Similar to CD patients, it was suggested that 4–5 principal components (each eigenvalue >1) provided 70.58-74.19% cumulative contribution of variance, as shown by the scree plot in Figure 1B. The four designed aggregating dimensions were identified as the explorative factor analysis demonstrated.

##### Discriminant validity

Independent *t*-test showed significantly higher total MCIBDQ and dimensional scores in “remission” group than “active” group in both CD and UC groups (all *P* < 0.001, Table 2), suggesting that MCIBDQ discriminated well between groups of CD and UC patients either in remission or with active disease.

##### Criterion validity

Moderate to high correlations (*r* ranged from 0.568–0.832) between MCIBDQ domains and SF-36 dimensions were found in this study, which was a perfect fit with the expectation of their correlations. (Table 2) Pearson correlations between “total MCIBDQ score and all domain scores” and total SF-36 score were significant, positive and significant (all *P* < 0.001), with coefficients ranging from 0.790 to 0.895 in patients with CD and from 0.747to 0.856 in patients with UC. Similarly, “total MCIBDQ score and all domain scores” for both CD (*r* ranged from −0.552 to −0.646) and UC (*r* ranged from −0.473 to −0.686) correlated negatively and significantly (all *P* < 0.001) with disease activity indices. Furthermore, significant correlations were also indicated between “total MCIBDQ score and all domain scores” and health transition on general health in both CD (all *P* < 0.001, *r* ranged from 0.526 to 0.596) and UC (*r* ranged from 0.476 to 0.531). No particular low correlation coefficient was detected as expected in the present study. (Tables 3 and 4)

**Table 3:** **Pearson correlation coefficients between MCIBDQ domains and SF-36 dimensions**

**SF-36 dimensions**	**MCIBDQ domains in CD**				**MCIBDQ domains in UC**			
	**Bowel symptoms**	**Systemic symptoms**	**Emotional function**	**Social function**	**Bowel symptoms**	**Systemic symptoms**	**Emotional function**	**Social function**
PF	.621	.666	.681	**.740**	.701	.659	.653	**.701**
RP	.607	.648	.692	**.712**	.704	.668	.728	**.728**
BP	**.724**	.750	**.741**	.776	**.673**	.675	**.652**	.615
GH	.666	**.710**	**.715**	.618	.499	**.568**	**.608**	.579
VT	**.724**	**.790**	**.832**	.740	.588	**.737**	**.708**	.653
SF	.634	.670	**.726**	**.746**	**.602**	.650	**.689**	**.653**
RE	.563	.594	**.686**	.570	.581	.638	**.729**	.628
MH	.664	.680	**.781**	.650	.502	.566	**.670**	.494

Pearson correlation coefficients in bold were those expected to be moderate or high.

All correlations were significant at the 0.001 level (2-tailed).

**Table 4:** **Pearson correlation coefficients between total MCIBDQ score and 4 domain scores and SF-36 total score, disease activity index and self-evaluation on general health score**

	**Total SF-36 score**	**DAI** ^**a**^	**Health transition**
MCIBDQ domains in CD			
Bowel symptoms	.790	-.646	-.527
Systemic symptoms	.846	-.580	-.596
Emotional function	.887	-.576	-.576
Social function	.826	-.552	-.526
Total MCIBDQ score	.895	-.617	-.586
MCIBDQ domains in UC			
Bowel symptoms	.747	-.686	-.507
Systemic symptoms	.810	-.473	-.519
Emotional function	.856	-.498	-.476
Social function	.780	-.580	-.524
Total MCIBDQ score	.847	-.604	-.531

^a^Disease activity index according to HBI or CAI.

All correlations were significant at the 0.001 level (2-tailed).

### The impact of different therapies on HRQOL

#### Before and after infliximab treatment

**Figure 2:**
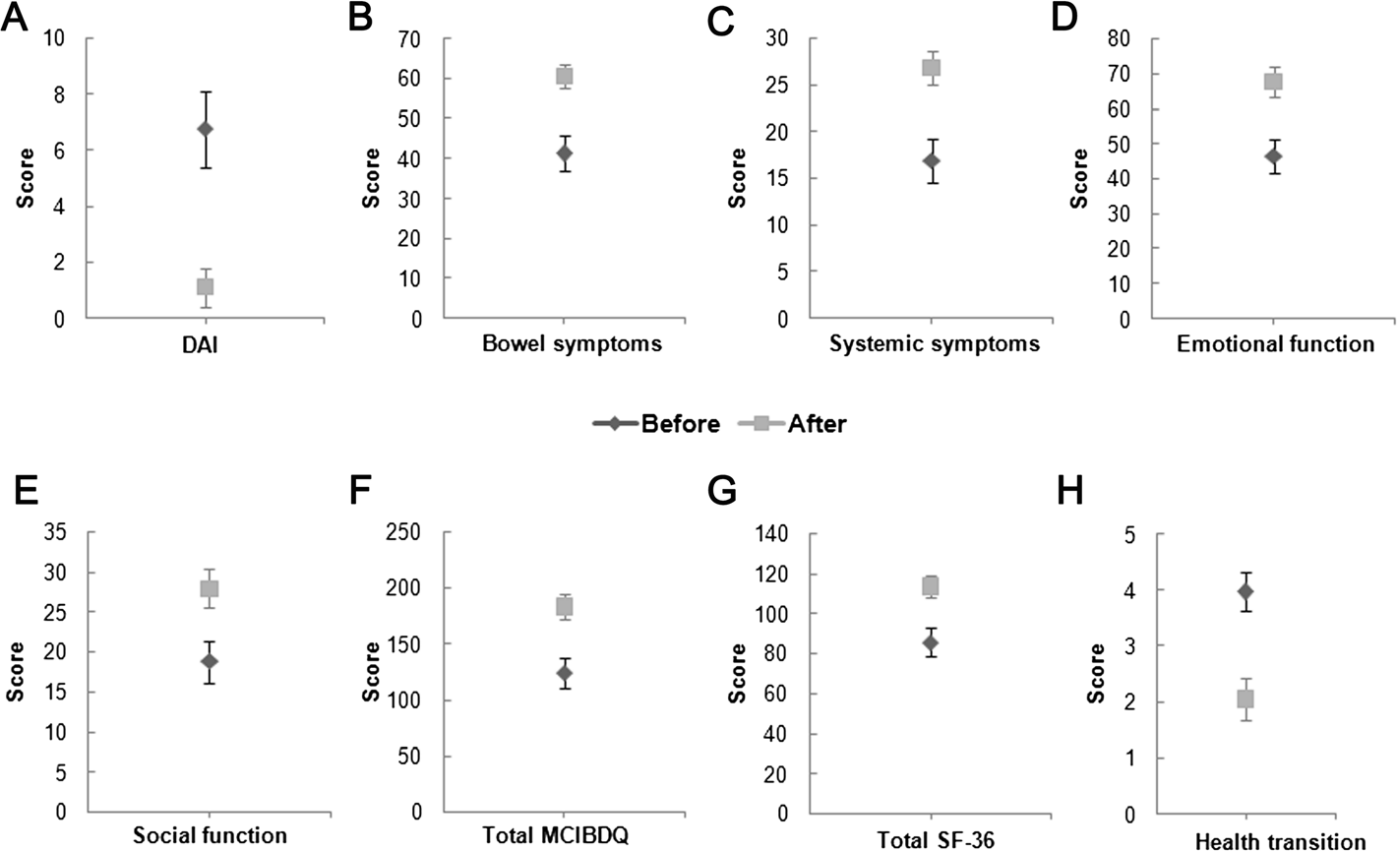
**The scores (including 95% CI) before and after infliximab treatment. A**, Disease activity index score; **B**, Bowel symptoms score; **C**, Systemic symptoms score; **D**, Emotional function score; **E**, Social function score; **F**, Total MCIBDQ score; **G**, Total SF-36 score; **H**, Health transition score.

In total, 41 participants (36 for CD and 5 for UC) with infliximab infusions completed the questionnaires before and after infliximab infusions. It was found that after infliximab treatment, DAI significantly decreased, total MCIBDQ score and all domain scores significantly increased, total SF-36 score significantly increased, and score of health transition on general health significantly decreased in patients with IBD (Figure 2 and see Additional file 4: Table S2]).

### The times of infliximab infusion

The comparison between the subgroups (*a*: 17 patients; *b*: 24 patients) showed that, the decrease of score of health transition on general health was significantly greater in group *b* versus group *a*. Moreover, compared with group *a*, there were trends that DAI decreased more and bowel symptoms domain score of MCIBDQ increased more in group *b* although it failed to reach statistical significance (see Additional file 4: Table S2).

### Comparisons among different therapies

**Figure 3:**
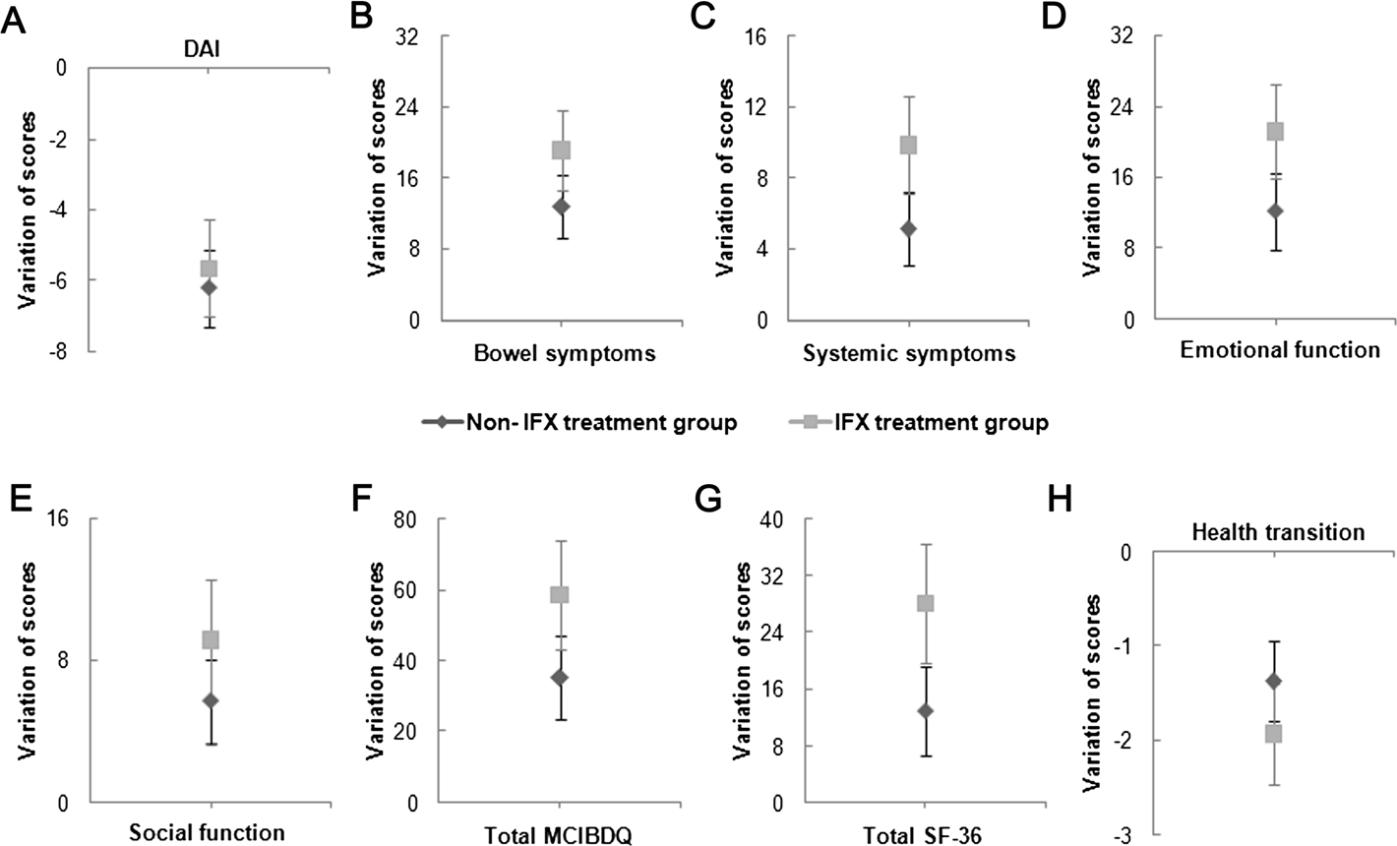
**Score variations (including 95% CI) of non-IFX treatment group and IFX treatment group. A**, Variation of disease activity index score; **B**, Variation of bowel symptoms score; **C**, Variation of systemic symptoms score; **D**, Variation of emotional function score; **E**, Variation of social function score; **F**, Variation of total MCIBDQ score; **G**, Variation of total SF-36 score; **H**, Variation of health transition score.

Compared with patients treated without infliximab (N = 63, 38 for CD and 25 for UC), the improvements of overall SF-36 scores, overall MCIBDQ score and most domain scores were significantly greater in infliximab group than those of the conventional treatment group. We found similar results in comparison between group 3 and group 4 (Figure 3 and see Additional file 4: Table S2.

### Comparisons between paired groups

**Figure 4:**
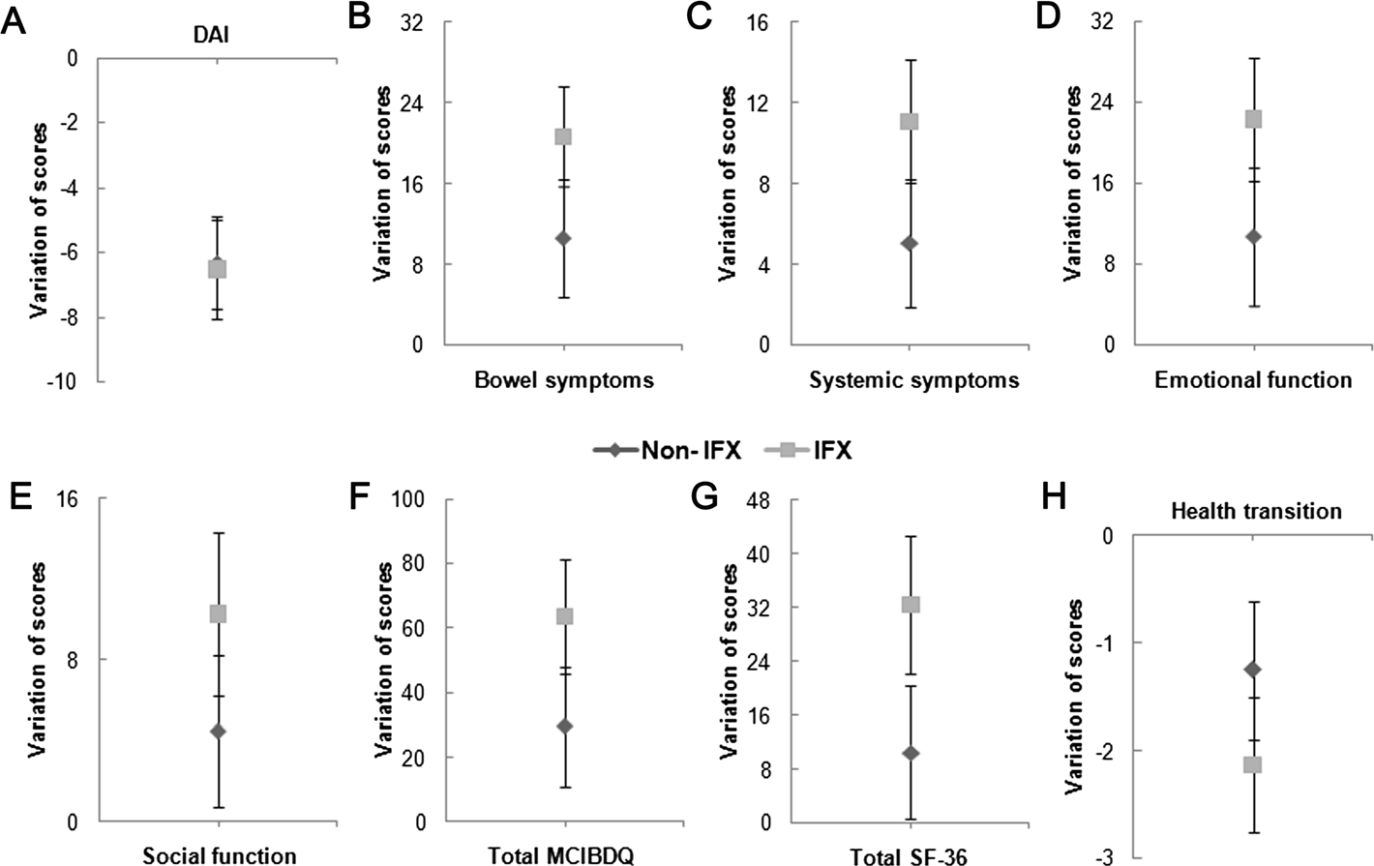
**Paired comparison between non-IFX treatment group and IFX treatment group (including 95% CI). A**, Variation of disease activity index score; **B**, Variation of bowel symptoms score; **C**, Variation of systemic symptoms score; **D**, Variation of emotional function score; **E**, Variation of social function score; **F**, Variation of total MCIBDQ score; **G**, Variation of total SF-36 score; **H**, Variation of health transition score.

Similar to the results above, paired comparison suggested overall SF-36 scores, overall MCIBDQ score and all domain scores increased more significantly in infliximab group than in placebo group. Furthermore, compared with control group, the score of health transition on general health decreased more in infliximab group. However, it only showed a trend without significance (Figure 4 and see Additional file 4: Table S2).

### Marriage, employment and economic burden

According to the results, infliximab treatment significantly reduced the negative impact on marriage (*P* = 0.037), increased work time (*P* = 0.016) and eased economic burden (*P* = 0.048) in IBD patients. Comparisons among different therapies showed work time increased significantly more in IFX group (*P* = 0.039). Furthermore, paired comparison suggested negative impact on love in IFX group reduced significantly more (*P* = 0.050). Detailed results were shown on Additional file 5: Table S3.

## Discussion

The measurement of HRQOL is not only an important approach in the clinical evaluation, but also in the therapeutic management of individual patients [31]. Thus, a reliable and valid instrument for HRQOL assessment is in urgent need currently. In the present study, MCIBDQ was demonstrated reliable and valid, with strong and significant correlation to SF-36 and DAI, and with moderate correlation to transition on general health. These results were similar both in CD and in UC patients, in line with the results of other language versions.

HRQOL including several domains can be impaired in many ways in IBD patients. In pace with the shifting of health concepts, patients consider the quality of life after being diagnosed and treated more, rather than barely dealing with the symptoms. Infliximab, as a safe and effective biological agent, was mainly used in patients with refractory CD and UC. The present study suggested that infliximab treatment significantly alleviate disease activity and improve HRQOL of IBD patients, in accordance with similar preceding studies [10,11]. Moreover, compared to patients without infliximab infusion and patients treated with immunosuppressor, HRQOL increased significantly more in patients with infliximab infusion. Probably due to the insufficient sample size of group 1 and 2, their comparisons to infliximab group did not show significant difference. To our knowledge, this was the first time the impact of different treatments on HRQOL was explored in China.

It was noteworthy that the improvement of DAI did not show significant difference between groups, which may be associated with the fact that DAI before treatment in infliximab group was not much greater than control group, but lower. As known, IFX is a huge economic burden for ordinary Chinese IBD patients. Since IFX treatment is not reimbursed in Mainland China, its administration is largely limited to relatively rich patients. On the other hand, regardless of DAI, the rich tend to have better healthcare and therapeutic effect after infliximab infusion, making DAI even lower in infliximab group and improve no greater than the control group, which is quite different from studies of other countries [7,10].

Many life aspects, such as marriage, employment and economic burden, can affect the quality of life in IBD patients. In this study, the social and cultural background might be the explanations. This study initially described the impact on “marriage, employment and economic burden” and assessed their changes after different treatments. Negative impact on marriage, employment and economic status was found in IBD patients.

After being diagnosed with IBD, many patients experienced a sense of inferiority, feeling embarrassed for the symptoms they suffered and receiving less understanding with regard to their social interactions and performance [32]. Moreover, long duration of awful experience brought them fatigue and heavy economic burdens, which placed a substantial impact on their marriage. Regarding employment, 32.97% lost their jobs because of the disease, which seemed to disagree with the working information of these subjects. The urgency of defecation or even incontinence is one prominent problem for IBD patients, compelling them to leave their original working environment where access to toilets was inconvenient [32]. Actually, high work pressure usually resulted in relapse for IBD patients, making their work time inevitably less. It was shown that infliximab treatment might help to reduce the negative impact on love and increase their work time, which was estimated to perform better than conventional therapies.

Interestingly, it was found that infliximab administration helped lighten the economic burden of IBD patients. In China, infliximab is not included in the Essential Medicine List (EML), neither covered by healthcare insurance provided by the government. As a result, only patients with high incomes would choose infliximab treatment, and they may not be as sensitive as low-income patients regarding medical cost, while they tend to pay more attention to efficacy. If it turns out to be effective, they are more inclined to be satisfied with the therapy and the cost. Maybe this partly explains the reduction of economic burden in patients who received infliximab infusions.

## Conclusion

MCIBDQ was demonstrated to be a reliable and valid scale applied in Chinese IBD patients. Infliximab treatment was found to significantly alleviate disease activity and to significantly improve HRQOL of IBD patients. Moreover, compared with patients without infliximab infusion, infliximab significantly increased HRQOL. Negative impact on marriage, employment and economic status was found in IBD patients. It was shown in the present study that infliximab treatment might help to reduce the negative impact on love, increase their work time and lighten their economic burden.

## Additional files

Additional file 1: Table S1. Details of MCIBDQ and Chinese version of SF-36. Additional file 2:
**Scale of marriage, employment and economic burden.** Detailed content of the “Scale of marriage, employment and economic burden” was shown in this file. Additional file 3:
**Supplementary methods.** The supplementary methods showed the background, methods and the detailed process of the validation of MCIBDQ. Additional file 4: Table S2. Comparison of HRQOL scores among different treatment groups. Additional file 5: Table S3. Comparison of scores in marriage, employment and economy among different treatment groups.

## Abbreviations

IBD: Inflammatory bowel disease
CD: Crohn’s disease
UC: Ulcerative colitis
HRQOL: Health related quality of life
IFX: Infliximab
TNF-α: Tumor necrosis factor-alpha
IBDQ: Inflammatory bowel disease questionnaire
MCIBDQ: Mainland Chinese Version of IBDQ
SF-36: Life-36-item short form health survey scale
HBI: Harvey-Bradshaw simple index
CAI: Walmsley simple colitis activity index
KMO: Kaiser-Meyer-Olkin
EML: Essential medicine list
