# Supplementary material for: Different therapeutic approaches on quality of life in patients with inflammatory bowel disease
Source: BMC Gastroenterol. 2014 Nov 25;14:199. doi: 10.1186/s12876-014-0199-5 (PMC4271410; doi:10.1186/s12876-014-0199-5)
Supplement: Additional file 1: Table S1. — Details of MCIBDQ and Chinese version of SF-36. [file 12876_2014_199_MOESM1_ESM.pdf]

Additional file 1

Supplementary tables

Supplementary Table 1.Details of MCIBDQ and Chinese version of SF-36

Dimension	Number of items	Score range	Items distribution
**MCIBDQ**			
Bowel symptoms	10	10-70	1+5+9+13+17+20+22+24+26+29
Systemic symptoms	5	5-35	2+6+10+14+18
Emotional function	12	12-84	3+7+11+15+19+21+23+25+27+30+31+32
Social function	5	5-35	4+8+12+16+28
**SF-36**			
PF	10	10-30	3a+3b+3c+3d+3e+3f+3g+3h+3i+3j
RP	4	4-8	4a+4b+4c+4d
BP	2	2-11	7+8
GH	5	5-25	1+11a+11b+11c+11d
VT	4	4-24	9a+9e+9g+9i
SF	2	2-10	6+10
RE	3	3-6	5a+5b+5c
MH	5	5-30	9b+9c+9d+9f+9h
Health transition	1	1-5	2

PF: Physical functioning, RP: Role limitations due to physical health problems, BP: Bodily pain, GH: General health perception, VT: Vitality, SF: Social functioning, RE: Role limitations due to emotional problems, MH: Mental health.
