# Supplementary material for: Different therapeutic approaches on quality of life in patients with inflammatory bowel disease
Source: BMC Gastroenterol. 2014 Nov 25;14:199. doi: 10.1186/s12876-014-0199-5 (PMC4271410; doi:10.1186/s12876-014-0199-5)
Supplement: Additional file 2: — Scale of marriage, employment and economic burden. Detailed content of the “Scale of marriage, employment and economic burden” was shown in this file. [file 12876_2014_199_MOESM2_ESM.pdf]

Additional file 2

Scale of marriage, employment and economic burden

Marriage:

1. After being diagnosed of IBD, to what extent has the disease affected your marriage or love life?

   ①Not at all ②Slightly ③Moderately ④Quite a bit ⑤Extremely

2. After being diagnosed of IBD, what do you think about your marital happiness?

   ①Poor ②Fair ③Good ④Very good ⑤Excellent

Employment:

3. After being diagnosed of IBD, to what extent has the disease affected your employment?

   ①Not at all ②Slightly ③Moderately ④Quite a bit ⑤Extremely

4. After being diagnosed of IBD, to what extent has the disease reduced your work time?

   ①Significantly ②Moderately ③Not at all

5. Did you lose your job because of IBD?

   ①Yes ②No

Economic burden:

6. After being diagnosed of IBD, to what extent has the disease laid economic burden on you or your family?

   ①Not at all ②Slightly ③Moderately ④Quite heavy ⑤Extremely heavy
