# Supplementary material for: Different therapeutic approaches on quality of life in patients with inflammatory bowel disease
Source: BMC Gastroenterol. 2014 Nov 25;14:199. doi: 10.1186/s12876-014-0199-5 (PMC4271410; doi:10.1186/s12876-014-0199-5)
Supplement: Additional file 3: — Supplementary methods. The supplementary methods showed the background, methods and the detailed process of the validation of MCIBDQ. [file 12876_2014_199_MOESM3_ESM.pdf]

# Additional file 3

## Supplementary methods

### *Validation of MCIBDQ*

Reliability

In general, the reliability of an instrument can be considered as the degree of consistency of its measurement [1], with two main forms: test-retest reliability and internal consistency. Test–retest reliability was assessed by the stable patients whose disease activity index (DAI) remained the same or changed no more than 1 point and completed the follow-up MCIBDQ, using the intra-class correlation coefficient (ICC). In the present study, however, insufficient stable patients (only 10 patients with CD and 5 with UC)made it unpractical. Internal consistency could be represented by Cronbach's α (values of >0.7 indicating adequate consistency).

Validity

For a medical or health care instrument to be clinically useful, it must possess validity. Validity, the ability of a instrument to assess fully what it was intended to measure, was assessed in 4 ways according to standard definitions including content validity, construct validity, discriminant validity, and criterion validity.[2,3]

Content validity, the extent to which the instrument accurately measured what it was purported to measure, was tested by experienced gastroenterologists about whether the MCIBDQ was an appropriate and clinically useful measure of HRQOL or not.

Construct validity, the extent to which a created instrument matched the theoretical construct and known or postulated features of a disease or condition, was tested by factor analysis.

Discriminant validity of an instrument represented the ability to distinguish between groups of subjects. In the present study, it referred to how well the MCIBDQ differentiates patients with active IBD from those in remission according to HBI or CAI scores. As was mentioned above, in both indices, $<4$ were indicated as quiescent and $\geq 4$ were considered as active. Independent samples *t*-test was used to assess the ability.

Criterion validity referred to the ability of an instrument to correlate positively and significantly with an established or accepted gold standard test. Actually, the accepted disease-specific gold standard instrument for patients with IBD hadn't been established yet. Thus, in this study, the correlations between MCIBDQ and widely accepted externally derived criteria including Chinese SF-36, HBI or CAI, and the single scale on health transition, were evaluated by testingthe following hypotheses which have been put forward by other authors [4]:

1) The IBDQ bowel domain was hypothesized to have a moderate correlation with the SF-36 dimensions BP and VT.

2) The IBDQ systemic domain was hypothesized to have a moderate to high correlation with the SF-36 dimensions VT and GH.

3) The IBDQ social domain was hypothesized to have a high correlation with the SF-36 dimension of SF, a moderate correlation with the SF-36 dimensions PF and RP.

4) The IBDQ emotional domain was hypothesized to have a high correlation with the SF-36 dimension of MH, a moderate correlation with the SF-36 dimensions RE, VT, BP, SF, and GH.

5) The IBDQ total score was hypothesized to have a moderate to high correlation with the SF-36 total score.

6) The IBDQ domain scores were hypothesized to have a moderate to high correlation with disease activity indices.

7) The IBDQ domain scores were hypothesized to have a low to moderate correlation with the global score for general well-being.

Pearson correlation coefficients were calculated to represent the correlations between domains and scales, and >0.5 was considered to be moderate correlation and >0.7 was considered as high correlation.

## References


1. Streiner DL, Norman GR. Reliability. In: *Health Measurement Scales. A Practical Guide to their Development and Use*, 2nd edn. New York: Oxford University Press, 1995:104-27.
2. Streiner DL, Norman GR. Validity. In: *Health Measurement Scales. A Practical Guide to their Development and Use*, 2nd edn. New York: Oxford University Press, 1995:144-62.
3. Ely EW, Truman B, Shintani A, et al. Monitoringsedation status over time in ICU patients: reliability and validity of theRichmond Agitation-Sedation Scale (RASS). *JAMA* 2003 Jun 11;289(22):2983-91.
4. Ren WH, Lai M, Chen Y, Irvine EJ, Zhou YX. Validation of the mainland Chinese version of

the Inflammatory Bowel Disease Questionnaire (IBDQ) for ulcerativecolitis and Crohn's disease. *Inflamm Bowel Dis* 2007 Jul;13(7):903-10.
