# Supplementary material for: Different therapeutic approaches on quality of life in patients with inflammatory bowel disease
Source: BMC Gastroenterol. 2014 Nov 25;14:199. doi: 10.1186/s12876-014-0199-5 (PMC4271410; doi:10.1186/s12876-014-0199-5)
Supplement: Additional file 4: Table S2. — Comparison of HRQOL scores among different treatment groups. [file 12876_2014_199_MOESM4_ESM.pdf]

Additional file 4

Supplementary Table 2. Comparison of HRQOL scores among different treatment groups

Treatment group	Non- IFX treatment group (level 1-3)				IFX treatment group (level 4)			Paired groups	
	1 (N=15)	2 (N=21)	3 (N=27)	Total (N=63)	<4 times[a] (N=17)	≥ 4times[a] (N=24)	Total (N=41)	Non-IFX (N=32)	IFX
**DAI**									
Before[b]	6.47	8.38	7.65	7.61	6.35	7.06	6.77	7.77	7.92
After[b]	1.53	1.17	1.52	1.40	2.09	0.42	1.11	1.42	1.39
Variation[b]	-4.93	-7.21	-6.13	-6.21	-4.26	-6.65	-5.66	-6.34	-6.53
*P*value[c]	0.577	0.194	0.655	0.528	0.081		**<0.001**	0.702	
**MCIBDQ**									
**Bowel symptoms**									
Before[b]	43.80	44.17	47.25	45.37	41.76	41.25	41.46	47.85	39.28
After[b]	56.16	60.70	57.69	58.32	56.27	63.71	60.63	58.64	59.93
Variation[b]	12.30	16.37	10.39	12.83	14.51	22.46	19.16	10.56	20.65
*P*value[c]	0.114	0.469	**0.019**	**0.029**	0.060		**<0.001**	**0.007**	
**Systemicsymptoms**									
Before[b]	19.60	19.05	18.81	19.08	15.12	18.17	16.90	19.72	15.53
After[b]	24.36	25.87	22.85	24.21	24.18	28.63	26.78	24.78	26.59
Variation[b]	4.50	6.82	4.15	5.15	9.06	10.46	9.88	5.06	11.06
*P*value[c]	**0.046**	0.189	**0.008**	**0.006**	0.615		**<0.001**	**0.007**	
**Emotional function**									
Before[b]	50.00	52.10	53.50	52.18	43.66	48.50	46.49	56.34	44.38
After[b]	59.73	67.84	64.30	64.47	60.35	72.88	67.68	67.09	66.72
Variation[b]	9.51	15.74	10.73	12.18	16.69	24.38	21.19	10.74	22.34
*P*value[c]	**0.028**	0.221	**0.022**	**0.010**	0.154		**<0.001**	**0.015**	
**Social function**									
Before[b]	21.77	20.76	22.03	21.54	17.42	19.70	18.79	22.98	17.40
After[b]	26.86	28.55	26.23	27.16	23.89	30.65	27.94	27.45	27.67
Variation[b]	5.23	7.79	4.29	5.71	6.47	10.95	9.16	4.47	10.27
*P*value[c]	0.196	0.640	0.054	0.086	0.192		**<0.001**	**0.024**	
**Total MCIBDQ**									
Before[b]	135.17	136.08	141.58	138.17	119.81	127.61	124.49	146.89	117.51
After[b]	167.10	181.77	171.07	173.67	164.18	195.85	183.19	177.09	181.05
Variation[b]	31.55	45.24	29.56	35.25	44.37	68.24	58.69	29.55	63.54
*P*value[c]	0.066	0.303	**0.019**	**0.015**	0.127		**<0.001**	**0.004**	
**Total SF-36**									
Before[b]	88.53	89.26	95.76	92.03	78.80	88.54	84.50	95.20	79.58

After[b]	104.21	110.32	101.45	104.95	103.25	120.39	113.80	105.21	113.14
Variation[b]	14.34	21.66	6.20	12.94	22.22	31.85	28.14	10.45	32.40
*P*value[c]	0.084	0.389	**0.001**	**0.004**	0.269		**<0.001**	**0.002**	
**Health transition**									
Before[b]	4.07	3.76	3.93	3.90	3.94	4.00	3.98	3.97	4.06
After[b]	2.73	2.33	2.59	2.54	2.94	1.42	2.05	2.72	1.94
Variation[b]	-1.33	-1.43	-1.33	-1.37	-1.00	-2.58	-1.93	-1.25	-2.13
*P*value[c]	0.273	0.298	0.125	0.102	**0.002**		**<0.001**	0.075	

a, according to the times of IFX infusion till the last interview, subjects in IFX group were divided into 2 subgroups:<4 timesand ≥ 4 times;

b, the mean scores before treatment or after treatment were calculated on all available data excluding the dropped domain scores; all the variations were defined as (score of the interview after treatment minus score of the interview before treatment) without dropped domains both before and after treatment;

c. Independent *t*-test and paired *t*-test were applied. *P* values in the groups of treatment level 1, 2 or 3 were assessed by comparing thescore variance between level 1 and level 4, level 2 and level 4 ,and level 3 and level 4, respectively; *P* values in the total non-IFX treatment group were assessed by comparing the score variance between thetotal non-IFX treatment group and the total IFX treatment group; *P* values in subgroups of IFX treatment group were assessed by comparing the score variance betweenthese two subgroups; *P* values in total IFX treatment group were assessed by comparing the score variance betweenscore of the 2nd interview and score of the 1st interview; *P* values in paired groups were assessed by comparing the score variance betweenthese two paired groups.

The data of missing responses were excluded according to setting of PASW Statistics 18.0. The *P* valuesin bold were those with significance at the 0.05 level (2-tailed).
