# Supplementary material for: Different therapeutic approaches on quality of life in patients with inflammatory bowel disease
Source: BMC Gastroenterol. 2014 Nov 25;14:199. doi: 10.1186/s12876-014-0199-5 (PMC4271410; doi:10.1186/s12876-014-0199-5)
Supplement: Additional file 5: Table S3. — Comparison of scores in marriage, employment and economy among different treatment groups. [file 12876_2014_199_MOESM5_ESM.pdf]

Additional file 5

Supplementary Table3. Comparison of scores in marriage, employment and economy among different treatment groups

Treatment group	Non- IFX treatment group (level 1-3)				IFX treatment group (level 4)			Paired groups		Total (N=104)
	1 (N=15)	2 (N=21)	3 (N=27)	Total (N=63)	<4 times[a] (N=17)	≥ 4times[a] (N=24)	Total (N=41)	Non- IFX (N=32)	IFX	
**Marriage**										
**Impact on love**										
Before[b]	2.54	2.12	2.25	2.33	3.33	2.87	3.10	2.18	3.32	2.57
After[b]	2.46	1.94	2.25	2.25	3.20	2.00	2.60	2.11	2.64	2.35
Variation[b]	-0.08	-0.18	0.00	-0.07	-0.13	-0.87	-0.50	-0.07	-0.68	-0.23
*P*value[c]	0.300	0.344	0.110	0.110	0.110		**0.037**	**0.050**		0.056
**Marital satisfaction**										
Before[b]	3.40	3.56	3.81	3.59	3.38	3.89	3.65	3.86	3.38	3.63
After[b]	3.40	3.81	3.69	3.67	3.25	4.00	3.65	3.76	3.46	3.66
Variation[b]	0.00	0.25	-0.13	0.05	-0.13	0.11	0.00	-0.10	0.08	0.03
*P*value[c]	1.000	0.310	0.618	0.826	0.510		1.000	0.279		0.727
**Employment**										
**Impact on employment**										
Before[b]	2.64	2.84	3.12	2.95	3.21	2.90	3.03	2.69	3.19	2.96
After[b]	2.93	2.68	2.88	2.83	2.93	2.35	2.59	2.55	2.62	2.74
Variation[b]	0.29	-0.16	-0.24	-0.09	-0.29	-0.55	-0.44	-0.14	-0.58	-0.22
*P*value[c]	0.061	0.430	0.543	0.149	0.583		0.066	0.335		0.070
**Work time**										
Before[b]	1.79	1.68	1.96	1.81	1.64	1.72	1.69	1.97	1.63	1.78
After[b]	1.93	1.89	1.76	1.84	1.86	2.22	2.06	1.86	2.00	1.92
Variation[b]	0.14	0.21	-0.20	0.02	0.21	0.50	0.38	-0.10	0.38	0.14
*P*value[c]	0.195	0.430	**0.008**	**0.039**	0.315		**0.016**	0.096		0.063
**Unemployment**										

Before[b]	1.64	1.70	1.56	1.63	1.64	1.83	1.75	1.66	1.75	1.67
**Economy**										
**Economic burden**										
Before[b]	3.33	3.30	3.73	3.52	4.12	3.30	3.65	3.52	3.78	3.55
After[b]	3.33	3.30	3.54	3.41	3.82	3.13	3.43	3.48	3.47	3.42
Variation[b]	0.00	0.00	-0.19	-0.08	-0.29	-0.17	-0.23	-0.03	-0.31	-0.14
*P*value[c]	0.133	0.185	0.865	0.303	0.597		**0.048**	0.130		**0.043**

a, according to the times of IFX infusion till the last interview, subjects in IFX group were divided into 2 subgroups: <4 times and ≥ 4 times;

b, the mean scores before treatment or after treatment were calculated on all available data excluding those didn't complete both before and after; all the variations were defined as (score of the interview after treatment minus score of the interview before treatment) without dropped domains both before and after treatment;

c. Independent t-test and paired t-test were applied. P values in the groups of treatment level 1, 2 or 3 were assessed by comparing the score variance between level 1 and level 4, level 2 and level 4 ,and level 3 and level 4, respectively; *P* values in the total non-IFX treatment group were assessed by comparing the score variance between the total non-IFX treatment group and the total IFX treatment group; *P* values in subgroups of IFX treatment group were assessed by comparing the score variance between these two subgroups; *P* values in total IFX treatment group were assessed by comparing the score variance between score of the 2nd interview and score of the 1st interview; *P* values in paired groups were assessed by comparing the score variance between these two paired groups.

The data of missing responses were excluded according to setting of PASW Statistics 18.0. The *P* values in bold were those with significance at the 0.05 level (2-tailed).
